# Tetrabutylammonium Bromide Media Aza-Michael Addition of 1,2,3,6-Tetrahydrophthalimide to Symmetrical Fumaric Esters and Acrylic Esters under Solvent-Free Conditions

**DOI:** 10.3390/molecules15107353

**Published:** 2010-10-21

**Authors:** Gholamhassan Imanzadeh, Farzaneh Ahmadi, Mohammadreza Zamanloo, Yagoub Mansoori

**Affiliations:** Department of Chemistry, College of Science, University of Mohaghegh Ardabili, 56199-11367, Ardabil, Iran

**Keywords:** aza-Michael addition, 1,2,3,6-tetrahydrophthalimide, fumaric ester, tetrabutyl-ammonium bromide

## Abstract

The aza-Michael addition of 1,2,3,6-tetrahydrophthalimide with symmetrical fumaric esters has been performed efficiently in a solvent-free system at 100 °C and using 1,4-diazabicyclo[2.2.2]octane (DABCO) as a base in the presence of tetrabutylammonium bromide (TBAB). The products were obtained in good to high yields within 2.5-7.0 h. This reaction worked well on linear alkyl fumarates and was not effective with nonlinear alkyl fumarates. Although the reaction was also applicable to acrylates such as *n*-butyl acrylate, methacrylates and crotonates were not suitable Michael acceptors for this reaction.

## 1. Introduction

Over the past decade, protection of the environment and waste prevention have been increasingly emphasized by researchers from both academia and industry [1]. For this reason, the elimination or reduce of volatile solvents in organic synthesis is a most important goal in green chemistry [2,3,4]. In this context the replacement of hazardous solvents with environmentally benign solvents or the development of solvent-free synthesis methods have become an important and popular research topic in recent years [5,6,7,8,9,10,11,12,13,14,15]. One of the most fundamental reactions in synthetic organic chemistry is the conjugate addition of nucleophilic species to the β-carbon of α,β-unsaturated systems. Because this process allows the construction of carbon backbones it is very valuable from a synthetic point of view [16]. Along this line, the aza-Michael addition reaction is widely recognized as one of the most important carbon-nitrogen bond–forming reaction in organic synthesis [17]. Most of the products of these reactions have special properties [18]. For instance the β-amino acids that can be obtained from aza-Michael additions between amines and α,β-unsaturated esters are attractive precursors in preparation of a variety of bioactive molecules such as taxol which is an anticancer drug in clinical use [19,20]. The Michael addition reactions of amines to α,β-unsaturated systems are usually carried out in the presence suitable catalysts, including silica-gel [21], ionic liquids [22], palladium [23], BiX_3_ (X=NO_3_, OTF) [24,25], pyrolidine-thiourea [26], Amberlyst-15 [27], and Cu-nanoparticles [28]. 

Neutral amides and imides have very restricted nucleophilicity, but under strong basic conditions, nitrogen anions derived from them can become more convenient nucleophiles in Michael-type additions to α,β-unsaturated compounds [29]. In a literature survey of the use of amides and imides as Michael donors, a few reports were found [30,31,32,33]. For these reasons, and in keeping with our ongoing program on the development of cleaner pathways, we recently reported the aza-Michael additions between phthalimide and alkyl or aryl acrylates in the presence of tetrabutylammonium bromide (TBAB) and 1,4-diazabicyclo[2.2.2]octane (DABCO) under solvent–free conditions [34]. We also studied the Michael addition of phthalimide to symmetrical fumaric esters in ionic liquid media [35]. 

**Scheme 1:**
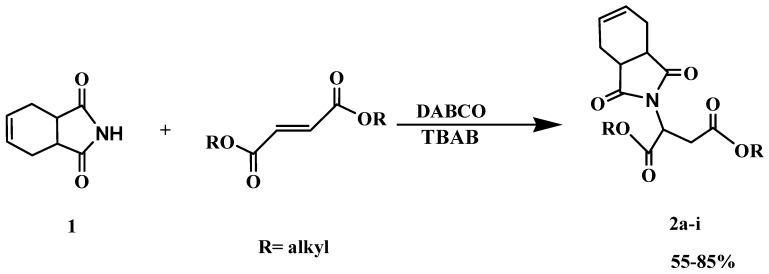
Aza-Michael addition of imide (**1**) to fumaric esters.

Herein, we report that aza-Michael addition of 1,2,3,6-tetrahydrophthalimide (**1**) to symmetrical fumaric esters in the presence of tetrabutylammonium bromide (TBAB) and 1,4-diazabicyclo[2.2.2]-octane (DABCO) provides the corresponding Michael adducts in good yields (Scheme 1). The reaction was carried out under solvent-free conditions at 100 °C with conventional heating. Both the ionic liquid used in our previous work [35], and the TBAB in the present work were found to be recyclable, however recovery of TBAB was easier than recovery of ionic liquid and the latter showed a slight decrease in catalytic activity do in this reaction we decided to replace the ionic liquid with TBAB.

## 2. Results and Discussion

As a first example, the addition of 1,2,3,6-tetrahydrophthalimide to diethyl fumarate was selected as a model reaction for the optimization reaction conditions and others parameters, such as nature of base and role of solvent. The obtained results of this typical reaction are presented in Table 1. From these results, it was observed that this reaction did not proceed in acetone under the reflux conditions (Table 1, entries 6-9) and other solvents like DMSO, DMF and acetonitrile provided lower yields under similar conditions (Table 1, entries 1-5 and 10-15). The results of Table 1 also show that under solvent-free conditions, in the presence of TBAB, bases like Na_2_CO_3_, K_2_CO_3_, triethylamine and pyridine all produced the desired product in low yield, while the use of DABCO as base afforded the desired products in excellent yields within a shorter reaction time (2.5 h) (Table 1, entry 20). The reaction did not proceed at all in the absence of solvent and TBAB (Table 1, entries 21-25). Therefore, the use of DABCO, in the presence of TBAB, under solvent-free conditions was selected as the optimal conditions for this model reaction because is advantageous thanks to the elimination of solvents while giving the desired product in excellent yield. 

**Table 1:** Influence of reaction parameters on the addition of 1,2,3,6-tetrahydrophthalimide to diethyl fumarate .

Entry	Solvent^a^	Base	Time (h)	Yield (%)
1	DMSO	Na_2_CO_3_	24	10
2	DMSO	K_2_CO_3_	24	15
3	DMSO	Triethylamine	24	10
4	DMSO	Pyridine	24	12
5	DMSO	DABCO	24	23
6	Acetone	Na_2_CO_3_	24	-
7	Acetone	K_2_CO_3_	24	-
8	Acetone	Triethylamine	24	-
9	Acetone	Pyridine	24	-
10	Acetone	DABCO	24	17
11	DMF	Na_2_CO_3_	24	10
12	DMF	K_2_CO_3_	24	10
13	DMF	Triethylamine	24	16
14	DMF	Pyridine	24	10
15	DMF	DABCO	24	20
16	TBAB^b^	Na_2_CO_3_	24	30
17	TBAB^b^	K_2_CO_3_	24	40
18	TBAB^b^	Triethylamine	24	25
19	TBAB^b^	Pyridine	24	18
20	TBAB^b^	DABCO	2:5	85
21	None^c^	Na_2_CO_3_	24	-
22	None^c^	K_2_CO_3_	24	-
23	None^c^	Triethylamine	24	-
24	None^c^	Pyridine	24	-
25	None^c^	DABCO	24	-

^a^ Reactions were carried out on 1.0 mmol scale of 1,2,3,6-tetrahydrophthalimide with 1.2 equiv of diethylfumarate in the presence of 1.0 equiv base under reflux conditions. ^b^ With 0.5 equiv of TBAB at 100 °C. ^c^ At 100 °C.

Encouraged by this initial result, the reaction was repeated using the various fumarates in TBAB medium and using DABCO as a base, in the absence of any solvent. The results are summarized in Table 2. The method worked well on linear alkyl fumarates (Table 2, entries 2, 4-11) but did not work on nonlinear alkyl fumarates (Table 2, entries 3, 12). Perhaps the steric factors are responsible for this selectivity.

**Table 2:** Michael additions of 1,2,3,6-tetahydrophthalimide to fumaric esters in thepresence of DABCO and TBAB.

Entry	Ester	Product	Time(h)	Yield(%)^a,b^
1	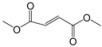	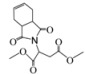	2.0	—
2	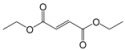	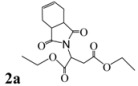	2.5	85
3	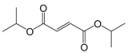	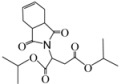	3.0	—
4	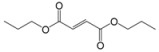	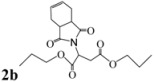	3.5	76
5	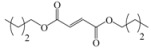	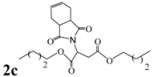	3.5	72
6	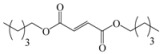	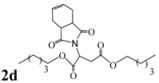	4.0	70
7	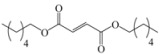	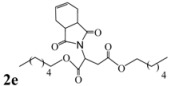	4.5	68
8	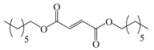	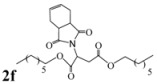	5.0	63
9	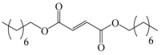	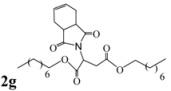	5.5	60
10	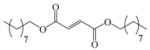	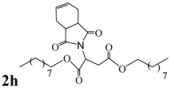	6.0	57
11	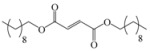	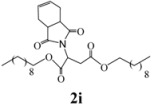	6.5	55
12	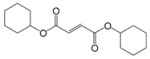	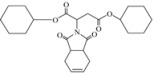	7.0	—
13	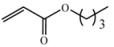	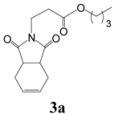	1.5	90
14	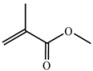	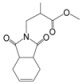	24.0	—
15	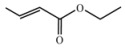	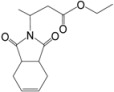	24.0	—

^a^ Isolated yield. ^b^ All products were novel and characterized by their ^ 1^H-NMR, ^13^C-NMR, IR, MS and elemental analysis data.

Our attempts to react methyl fumarate with Michael donor **1** under the model reaction conditions was unsuccessful (Table 2, entry 1). The reaction gave fumaric acid and imide **1 **without the formation of any Michael adduct. We believe that methyl fumarate is more susceptible to hydrolysis under the reaction conditions, due to its smaller groups. The reaction was also successfully applied for the addition of alkyl acrylates such as *n*-butyl acrylate which gave a 90% yield of the corresponding Michael adduct under the model reaction conditions (Table 1, entry 13). However, it was observed that sterically hindered α,β-unsaturated esters, like methyl methacrylate and ethyl crotonate, were not suitable Michael acceptors for this reaction and the starting materials were completely recovered after 24 h under the model reaction conditions (Table 2, entries 14, 15).

## 3. Conclusions

We have developed an efficient, simple and new method for aza-Michael addition of 1,2,3,6-tetrahydrophthalimide to symmetrical fumarates in the presence of TBAB under solvent-free conditions. We found that DABCO is the most suitable base for this reaction. It was found that this method selectively works on linear alkyl fumarates and did not occur with non linear alkyl fumarates as well as methyl methacrylate and ethyl crotonoate. Our studies showed that when TBAB was eliminated or replaced with a solvent, no reaction is observed. 

## 4. Experimental

### 4.1. General

TBAB was purchased from Fluka and DABCO, 1,2,3,6-tetrahydrophthalimide, fumaric acid and alcohols were purchased from Merck. All alkyl fumarates were synthesized in our laboratory according to the literature procedure [36] and their structures were confirmed by IR and ^1^H-NMR spectroscopy. The progress of the reactions was followed by TLC using silica gel SILIG/UV 254 plates. ^1^H-NMR (300 MHz) and ^13^C-NMR (75 MHz) spectra were recorded on a Bruker 300 MHz instrument. IR spectra were recorded on a Buck-Scientific 500 instrument. Mass spectra were recorded on a Shimadzu GC-MS-QP 1000PX. Elemental analysis for C, H, and N were performed using a Heraeus CHN-O-Rapid analyzer. The melting points were determined in open capillaries with a Stuart Melting Point Apparatus and are uncorrected. 

### 4.2. General procedure for Michael addition of 1,2,3,6-tetrahydrophthalimide to symmetrical fumaric esters

A mixture of 1,2,3,6-tetrahydrophthalimide (1.0 mmol), DABCO (1.0 mmol), TBAB (0.5 mmol), and fumaric ester (1.2 mmol) was kept at 100 ˚C in an oil bath for the stipulated time (Table 2). The progress of the reaction was monitored by TLC. After completion of the reaction, the reaction mixture was cooled to room temperature and dissolved in chloroform (40 mL). TBAB was recovered by the addition of water (15 mL), then collected and dried under vacuum. The chloroform layer was washed with water (3 × 15 ml). The organic layer was dried over anhydrous Na_2_SO_4._ The solvent was removed under reduced pressure and the resulting crude material was purified on short silica-gel column with ethyl acetate/*n*-hexane (2:8) as the eluent.

*Diethyl 2-(3a,4,7,7a-tetrahydro-1,3-dioxo-1H-isoindol-2(3H)-yl)succinate* (**2a**): Colorless solid; mp 45-47 ˚C; IR ν_max_(KBr)/cm^-1^ 2952, 1726, 1458, 985. ^1^H-NMR (CDCl_3_) δ ppm: 5.79-5.80 (m, 2H), 5.04 (dd, *J =* 8.40 Hz and 6.00 Hz, 1H ) 3.97-4.13 (m, 4H), 3.12 (dd, *J =* 16.50 Hz and 6.00 Hz, 1H), 3.03-3.05 (m, 2H), 2.78 (dd, *J =* 16.50 Hz and 8.40 Hz, 1H ), 2.44-2.50 (m, 2H), 2.13-2.20 (m, 2H), 1.09-1.21 (m, 6H). ^13^C-NMR (CDCl_3 _) δ ppm: 14.02, 23.28, 33.26, 40.21, 48.76, 60.85, 62.19, 127.38, 167.54, 169.75, 178.84. MS, *m/z* (%): 324 (37.0, M^+^ + 1), 277 (61.0), 249 (55.0), 203 (100.0), 177 (8.0), 99 (54.0), 79 (91.0), 70 (24.0), 43 (64.0). Anal. calcd for C_16_H_21_NO_6_: C 59.45, H 6.5, N 4.33; found C 59.71, H 6.34, N 4.69.

*Dipropyl 2-(3a,4,7,7a-tetrahydro-1,3-dioxo-1H-isoindol-2(3H)-yl)succinate* (**2b**): Colorless solid; mp 40-41 ˚C; IR ν_max_(KBr)/cm^-1^ 2955, 1739, 1427, 985. ^1^H-NMR (CDCl_3_) δ ppm: 5.78-5.80 (m, 2H). 5.08 (dd, *J =* 8.40 Hz and 6.35 Hz, 1H), 3.88-4.03 (m, 4H), 3.14 (dd, *J =* 16.64 Hz and 6.35 Hz, 1H), 3.02-3.04 (m, 2H), 2.80 (dd, *J =* 16.64 Hz and 8.40 Hz, 1H ), 2.44-2.51 (m, 2H), 2.14-2.20 (m, 2H), 1.47-1.59 (m, 4H), 0.77-0.88 (m, 6H).^ 13^C-NMR (CDCl_3_) δ ppm: 10.20, 21.78, 23.22, 33.31, 38.90, 48.89, 66.55, 67.66, 127.42, 167.67, 169.90, 178.85. MS, *m/z* (%): 352 (100.0, M^+^ + 1 ), 291 (78.5), 363 (26.0), 249 (86.50), 221 (69.0), 203 (83.0), 177 (40.50), 113 (23.50), 79 (87.0), 43 (84.0). Anal. calcd for C_18_H_25_NO_6_: C 61.52, H 7.17, N 3.99; found C 61.15, H 6.92, N 4.22.

*Dibutyl 2-(3a,4,7,7a-tetrahydro-1,3-dioxo-1H-isoindol-2(3H)-yl)succinate* (**2c**): Yellow oil; IR ν_max_(neat)/cm^-1^ 2843, 1727, 1460, 973. ^1^H-NMR (CDCl_3_) δ ppm: 5.82-5.89 (m, 2H), 5.54 (dd, *J =* 8.48 and 6.24 Hz, 1H), 3.98-4.08 (m, 4H), 3.77 (dd, *J =* 16.80 Hz and 6.24 Hz, 1H), 3.04-3.09 (m, 4H), 2.83 (dd, *J =* 16.80 Hz, 8.48 Hz, 1H), 2.48-2.55 (m, 2H), 2.18-2.23 (m, 2H), 1.49-1.55 (m, 4H), 1.25-1.31 (m, 4H), 0.83-0.88 (m, 6H).^ 13^C-NMR (CDCl_3_) δ ppm: 13.62, 18.97, 23.32, 30.45, 33.34, 38.92, 48.92, 64.89, 66.01, 127.45, 167.70, 169.49, 178.88. MS, *m/z* (%): 379 (17.0, M^+^), 305 (26.0), 249 (100.0), 221 (64.0), 203 (32.0), 177 (24.0), 79 (47.0), 57 (22.0), 41 (30.0). Anal. calcd for C_20_H_29_NO_6_: C 63.31, H 7.70, N 3.69; found C 63.58, H 7.12, N 3.91.

*Dipentyl 2-(3a,4,7,7a-tetrahydro-1,3-dioxo-1H-isoindol-2(3H)-yl)succinate* (**2d**): Pale yellow oil; IR ν_max_(neat)/cm^-1^ 2924, 1714, 1450, 985. ^1^H-NMR (CDCl_3_) δ ppm: 5.85-5.86 (m, 2H), 5.14 (dd, *J =* 8.48 Hz and 6.30 Hz, 1H), 4.00-4.10 (m, 4H), 3.20 (dd, *J =* 16.50 Hz and 6.30 Hz, 1H), 3.05-3.08 (m, 2H), 2.87 (dd, 1H, *J =* 16.50 Hz and 8.48 Hz), 2.52-2.57 (m, 2H), 2.20-2.27 (m, 2H), 1.53-1.60 (m, 4H), 1.24-1.30 (m, 8H), 0.84- 0.88 (m, 6H).^ 13^C-NMR (CDCl_3_) δ ppm: 17.80, 22.20, 23.24, 28.03, 33.31, 38.90, 48.87, 65.14, 66.25, 127.40, 167.67, 169.91, 178.82. MS, *m/z* (%): 408 (80.0, M^+^ + 1), 319 (24.0), 249 (100.0), 221 (56.0), 203 (26.3), 177 (26.0), 79 (48.5), 43 (53.0), 41 (21.0). Anal. calcd for C_22_H_33_NO_6_: C 64.83, H 8.16, N 3.44; found C 64.53, H 7.91, N 3.82.

*Dihexyl 2-(3a,4,7,7a-tetrahydro-1,3-dioxo-1H-isoindol-2(3H)-yl)succinate* (**2e**): Yellow oil; IR ν_max_(neat)/cm^-1^ 2943, 1727, 1472, 985. ^1^H-NMR (CDCl_3_) δ ppm: 5.81-5.82 (m, 2H), 5.10 (dd, *J =* 8.46 Hz and 6.28 Hz, 1H), 3.96-4.08 (m, 4H), 3.16 (dd, *J =* 16.0 Hz and 6.28 Hz, 1H), 3.04-3.05 (m, 2H), 2.82 (dd, *J =* 16.0 Hz and 8.46 Hz, 1H), 2.21-2.22 (m, 2H), 2.90-2.11 (m, 2H), 1.48-1.54 (m, 4H), 1.21-1.30 (m, 12H), 0.80-0.87 (m, 6H).^ 13^C-NMR (CDCl_3_) δ ppm: 20.00, 20.12, 23.32, 25.40, 28.30, 30.40, 31.45, 33.33, 38.91, 48.90, 65.01, 66.15, 127.35, 167.70, 169.94, 178.85. MS, *m/z* (%): 435 (12.0, M^+^), 305 (8.0), 249 (100.0), 221 (68.0), 203 (33.5), 177 (29.5), 79 (71.0), 57 (29.0), 43 (63.0), 41 (45.5). Anal. calcd for C_24_H_37_NO_6_: C 66.18, H 8.56, N 3.22; found C 66.23, H 8.18, N 3.61.

*Diheptyl 2-(3a,4,7,7a-tetrahydro-1,3-dioxo-1H-isoindol-2(3H)-yl)succinate* (**2f**): Pale yellow oil; IR ν_max_(neat)/cm^-1^ 2915, 1711, 1462, 976. ^1^H-NMR (CDCl_3_) δ ppm: 5.86-5.87 (m, 2H), 5.15 (dd, *J =* 8.70 Hz and 6.30 Hz, 1H), 4.01-4.11 (m, 4H), 3.21 (dd, *J =* 16.80 Hz and 6.30 Hz, 1H), 3.10-3.18 (m, 2H), 2.88 (dd, *J =* 16.80 Hz and 8.70 Hz, 1H), 2.53-2.59 (m, 2H), 2.21-2.28 (m, 2H), 1.53-1.58 (m, 4H), 1.23-1.29 (m, 16H), 0.84-0.86 (m, 6H).^ 13^C-NMR (CDCl_3_) δ ppm: 18.01, 23.34, 25.72, 28.60, 31.65, 33.31, 38.90, 48.88, 65.18, 66.29, 127.39, 127.48, 167.68, 169.92, 178.83. MS, *m/z* (%): 464 (35.0, M^+^ + 1), 347 (9.0), 249 (100.0), 221 (40.0), 204 (14.0), 177 (15.0), 79 (26.0), 57 (51.0), 43 (27.0). Anal. calcd for C_26_H_41_NO_6_: C 67.36, H 8.91, N 3.03; found C 67.82, H 9.14, N 3.78.

*Dioctyl 2-(3a,4,7,7a-tetrahydro-1,3-dioxo-1H-isoindol-2(3H)-yl)succinate* (**2g**): Pale yellow oil; IR ν_max_(neat)/cm^-1^ 2925, 1739, 1465, 973. ^1^H-NMR (CDCl_3_) δ ppm: 5.87 -5.89 (m, 2H), 5.17 (dd, , *J =* 8.70 Hz and 6.00 Hz, 1H), 4.02-4.12 (m, 4H), 3.23 (dd, *J =* 18.00 and 6.00 Hz, 1H), 3.11-3.17 (m, 2H), 2.90 (dd, *J =* 18.00 Hz, 8.70 Hz, 1H), 2.56-2.60 (m, 2H), 2.22-2.29 (m, 2H), 1.55-1.59 (m, 4H), 1.25-1.27 (m, 20H), 0.85-0.90 (m, 6H).^ 13^C-NMR (CDCl_3 _) δ ppm: 14.06, 22.61, 25.69, 25.81, 23.20, 25.75, 29.10, 31.74, 33.33, 38.90, 48.91, 65.25, 127.45, 167.70, 169.94, 178.84. MS, *m/z* (%): 492 (39.5, M^+^ + 1), 380 (5.0), 361 (8.0), 249 (100.0), 221 (36.0), 204 (12.5), 177 (13.0), 71 (21.0), 57 (23.5), 43 (29.9). Anal. calcd for C_28_H_45_NO_6_: C 68.91, H 9.23, N 2.85; found C 68.61, H 9.18, N 2.65.

*Dinonyl 2-(3a,4,7,7a-tetrahydro-1,3-dioxo-1H-isoindol-2(3H)-yl)succinate* (**2h**): Yellow oil; IR ν_max_(neat)/cm^-1^ 2925, 1745, 1450, 973. ^1^H-NMR (CDCl_3_) δ ppm: 5.83-5.88 (m, 2H), 5.12 (dd, *J =* 8.40 Hz and 6.20 Hz, 1H), 3.97-4.10 (m, 4H), 3.17 (dd, *J =* 16.70 Hz, 6.20 Hz, 1H), 3.00-3.06 (m, 2H), 2.87 (dd, *J =* 16.70 Hz and 8.40 Hz, 1H), 2.50-2.52 (m, 2H), 2.19-2.24 (m, 2H), 1.50-1.56 (m, 4H), 1.21-1.32 (m, 24H), 0.81-0.89 (m, 6H). ^13^C-NMR (CDCl_3_) δ ppm: 19.10, 23.34, 25.69, 25.89, 28.37, 29.14, 29.17, 31.61, 33.33, 38.90, 48.92, 65.18, 66.29, 127.40, 167.68, 169.91, 178.78. MS, *m/z* (%): 520 (17.0, M^+^ + 1), 305 (15.3), 249 (100.0), 221 (51.0), 203 (29.0), 177(26.0), 79(52.0), 57(40.0), 41(49.0).Anal. calcd for C_30_H_49_NO_6_: C 69.33, H 9.50, N 2.70; found C 69.74, H 9.12, N 2.91.

*Didecyl 2-(3a,4,7,7a-tetrahydro-1,3-dioxo-1H-isoindol-2(3H)-yl)succinate* (**2i**): Pale yellow oil; IR ν_max_(neat)/cm^-1^ 2925, 1733, 1460, 979. ^1^H-NMR (CDCl_3_) δ ppm: 5.86-5.87 (m, 2H), 5.15 (dd, *J =* 8.40 Hz and 6.30 Hz, 1H), 4.01-4.11 (m, 4H), 3.21 (dd, *J =* 16.65 Hz and 6.30 Hz, 1H ), 3.07-3.09 (m, 2H), 2.89 (dd, *J =* 16.65 Hz and 8.40 Hz, 1H), 2.53-2.58 (m, 2H), 2.21-2.28 (m, 2H), 1.56-1.58 (m, 4H), 1.24-1.30 (m, 28H), 0.84-0.88 (m, 6H).^ 13^C-NMR (CDCl_3_) δ ppm: 17.04, 21.15, 27.10, 28.47, 29.14, 29.40, 31.20, 31.84, 32.78, 33.32, 38.87, 48.91, 59.49, 62.98, 65.17, 66.27, 127.40, 167.66, 169.90, 178.79. MS, *m/z* (%): 548 (42.3, M^+^ + 1), 408 (6.6), 389 (8.0), 249 (100.0), 221 (31.5), 204 (11.0), 177 (13.0), 71 (19.0), 57 (33.0), 43 (37.0). Anal. calcd for C_32_H_53_NO_6_: C 70.17, H 9.75, N 2.56; found C 70.85, H 9.26, N 2.24.

*Dipropyl 3-(3a,4,7,7a-tetrahydro-1,3-dioxo-1H-isoindol-2(3H)-yl)propanoate* (**3a**): Colorless oil; IR ν_max_(neat)/cm^-1^ 2949, 1727, 1447, 979. ^1^H-NMR (CDCl_3_) δ ppm: 5.75-5.76 (m, 2H). 3.93 (d, *J =* 6.6 Hz, 2H) 3.63 (t, *J =* 7.20 Hz, 2H), 2.96-2.98 (m, 2H), 2.41-2.48 (m, 4H), 2.10 (t, *J =* 7.20 Hz, 2H), 1.43-1.52 (m, 2H), 1.19-1.31 (m, 2H), 0.78-0.83 (m, 3H).^ 13^C-NMR (CDCl_3_) δ ppm: 13.54, 18.95, 23.35, 30.41, 31.98, 34.46, 38.91, 64.50, 127.56, 170.51, 179.56. MS, *m/z* (%): 280 (18.0, M^+^ + 1), 279 (11.0), 205 (100.0), 177 (13.0), 163 (14.0), 99 (15.0), 79 (37.0), 55 (31.0). Anal. calcd for C_15_H_21_NO_4_: C 64.50, H 7.58, N 5.02; found C 64.87, H 7.85, N 5.61.

## References

[1] Anastas P.T., Warner J.C. (1998). Green Chemistry, Theory and Practice.

[2] Polshettiwar V., Varma R. S. (2008). Microwave-assisted organic synthesis and transformations using benign reaction media. Acc. Chem. Res..

[3] Polshettiwar V., Varma R. S. (2008). Aqueous microwave chemistry: A clean and green synthetic tool for rapid drug discovery. Chem. Soc. Rev..

[4] Toda F., Tanaka K. (2000). Solvent-free organic synthesis. Chem. Rev..

[5] Li C.J., Chen L. (2006). Organic chemistry in water. Chem. Soc. Rev..

[6] Ranu B.C., Banerjee S. (2007). Significant rate acceleration of the aza-Michael reaction in water. Tetrahedron Lett..

[7] Sharma G., Kumar R., Chakraborti A.K. (2008). On water’ synthesis of 2,4-diaryl-2,3-dihydro-1,5- benzothiazepines catalysed by sodium dodecyl sulfate (SDS). Tetrahedron Lett..

[8] Khatik G.L., Kumar R., Chakraborti A.K. (2006). Catalyst-free conjugated addition of thiols to α,β-unsaturated carbonyl compounds in water. Org. Lett..

[9] Sheldon R. (2001). Catalytic reactions in ionic liquids. Chem. Commun..

[10] Chankeshwara S.V., Chakraborti A.K. (2006). Catalyst-free chemoselective *N*-*tert*-butyloxycarbonylation of amines in water. Org. Lett..

[11] Luo Z.Y., Zang Q. S., Oderaotoshi Y., Curran D.P. (2001). Fluorous mixture synthesis: A fluorous-tagging strategy for the synthesis and separation of mixtures of organic compounds. Science.

[12] Harvath I.T. (1998). Fluorous biphase chemistry. Acc. Chem. Res..

[13] Oakes R.S., Califforrd A.A., Rayner C.M. (2001). The use of supercritical fluids in synthetic organic chemistry. J. Chem. Soc., Perkin Trans. I.

[14] Chen J., Spear S.K., Huddleston J.G., Rogers R.D. (2005). Aqueous polyethylene glycol solutions as green reaction media. Green Chem..

[15] Zhang Z.H., Yin L., Wang Y.M., Liu J.Y., Li Y. (2004). Indium tribromide in poly (ethylene glycol)(PEG): a novel and efficient recycle system for chemoselective deprotection of 1, 1-diacetates. Green Chem..

[16] Perlmutter P. (1992). Conjugate Addition Reactions in Organic Synthesis.

[17] Hayashi Y., Rohde J.J., Corey E.J. (1996). A novel super-Lewis acidic catalyst for enantioselective synthesis. J. Am. Chem. Soc..

[18] Kummaraja M., Pitchumani K.  (2006). Hetero-Michael addition of benzenethiol to cycloalkenones using cation-exchanged faujasites: simultaneous acid–base bifunctional catalysis. J. Mol. Catal. A: Chem..

[19] Vicario J.L., Badia D., Carrillo L. (2001). Asymmetric synthesis of β-substituted α-methyl-β-amino esters by mannich reaction of (*S*,*S*)-(*+*)-pseudoephedrine acetamide derived enolate with imine. Org. Lett..

[20] Gellman S. (1998). Foldmers: A manifesto. Acc. Chem. Res..

[21] Basu B., Das P., Hossain I. (2004). Synthesis of β-amino esters via aza-Michael addition of amines to alkenes promoted on silica: A useful and recyclable surface. Synlett.

[22] Yang L., Xu L.W., Zhou W., Li L., Xia C.G.  (2006). Highly efficient aza-Michael reactions of aromatic amines and *N*-heterocycles catalyzed by a basic ionic liquid under solvent-free conditions. Tetrahedron Lett..

[23] Kawatsura M., Hartwig J. F.  (2001). Transition metal-catalyzed addition of amines to acrylic acid derivatives. A high-throughput method for evaluating hydroamination of primary and secondary alkylamines. Organometallics.

[24] Srivastava N., Banik B. K. (2003). Bismuth nitrate-catalyzed versatile Michael reactions. J. Org. Chem..

[25] Varala R., Alam M.M., Adapa S.R. (2003). Michael type addition of aliphatic amines to α,β-ethylenic compounds using bismuth triflate catalyst. Synlett.

[26] Cao Y.J., Lai Y.Y., Wang X., Li Y.J., Xiao W.J. (2007). Michael additions in water of ketones to nitroolefins catalyzed by readily tunable and bifunctional pyrrolidine–thiourea organocatalysts. Tetrahedron Lett..

[27] Steves A.P., Silva M.E., Rodrigues L.M., Oliveria-Campos A.M.F., Hrdina R. (2007). Aza-Michael reactions with vinyl sulfones and Amberlyst-15 as catalyst. Tetrahedron Lett..

[28] Verma A.K., Kumar R., Chaudhary P., Saxena A., Shankar R., Mozumdar S., Chandra R. (2005). Cu-nanoparticles: A chemoselective catalyst for the aza-Michael reactions of *N*-alkyl- and *N*-arylpiperazines with acrylonitrile. Tetrahedron Lett..

[29] Mariella R., Jonauskas R. (1958). Cyanoethylation of aromatic amides. J. Org. Chem..

[30] Maggini M., Prato M., Ranelli M., Scorrano G. (1992). Synthesis of (−)-8-deoxy-7-hydroxy-swainsonine and (±)-6,8-dideoxy-castanospermine. Tetrahedron Lett..

[31] Corriu R.J.P., Oerz R. (1985). 1,4-Addition reactions to methacrylamide : A one pot synthesis of 3,4-dihydro 2(1H)-pyridinones and 3,5-disubstituted glutarimides. Tetrahedron Lett..

[32] Bredereck H., Gompper R., Herlinger H., Wotiun E. (1960). Säureamid-Reaktionen, XXIV. umsetzungen von formamid mit Mannich-basen. Chem. Ber..

[33] Reitz A., Verlander M., Goodman M. (1982). Alumina catalyzed transformations of *O*-(3-oxobutyl) urethanes. Tetrahedron Lett..

[34] Imanzadeh G.H., Khalafinezhad A., Zare A., Hasaninejad A., Mosavi Zare A., Parhami A. (2007). Michael addition of phthalimid and saccharin to α,β-unsaturated esters under solvent-free conditions. J. Iran. Chem. Soc..

[35] Imanzadeh I.G., Tavana M.M., Zamanloo M.R., Mansoori Y. (2009). Aza-Michael addition of isatin and phthalimide to symmetrical fumaric esters in ionic liquid media. Chin. J. Chem..

[36] Vogel A. (1978). Vogel^,^s Practical Organic Chemistry.

